# Anticancer Therapy–Related Increases in Arterial Stiffness: A Systematic Review and Meta‐Analysis

**DOI:** 10.1161/JAHA.119.015598

**Published:** 2020-07-10

**Authors:** Shannon K. Parr, Jia Liang, Keri L. Schadler, Susan C. Gilchrist, Catherine C. Steele, Carl J. Ade

**Affiliations:** ^1^ Department of Kinesiology College of Health and Human Sciences Kansas State University Manhattan KS; ^2^ Department of Statistics Kansas State University Manhattan KS; ^3^ Division of Pediatrics Department of Pediatrics The University of Texas MD Anderson Cancer Center Houston TX; ^4^ Department of Clinical Cancer Prevention and Department of Cardiology The University of Texas MD Anderson Cancer Center Houston TX; ^5^ Department of Food, Nutrition, Dietetics, Health Kansas State University Manhattan KS

**Keywords:** arterial stiffness, cancer therapy, cardiotoxicity, pulse wave velocity, vascular toxicity, Imaging

## Abstract

**Background:**

Cardio‐oncology is a clinical discipline focused primarily on the early detection of anticancer therapy–related cardiomyopathy. However, there is growing evidence that the direct adverse consequences extend beyond the myocardium to affect the vasculature, but this evidence remains limited. In addition, there remains a paucity of clinically based strategies for monitoring vascular toxicity in these patients. Importantly, arterial stiffness is increasingly recognized as a surrogate end point for cardiovascular disease and may be an important vascular outcome to consider. Therefore, the aim of this systematic review and meta‐analysis was to summarize evidence of increased arterial stiffening with anticancer therapy and evaluate the effect of treatment modifiers.

**Methods and Results:**

A total of 19 longitudinal and cross‐sectional studies that evaluated arterial stiffness both during and following anticancer therapy were identified using multiple databases. Two separate analyses were performed: baseline to follow‐up (12 studies) and control versus patient groups (10 studies). Subgroup analysis evaluated whether stiffness differed as a function of treatment type and follow‐up time. Standard mean differences and mean differences were calculated using random effect models. Significant increases in arterial stiffness were identified from baseline to follow‐up (standard mean difference, 0.890; 95% CI, 0.448–1.332; *P*<0.0001; mean difference, 1.505; 95% CI, 0.789–2.221; *P*≤0.0001) and in patient versus control groups (standard mean difference, 0.860; 95% CI, 0.402–1.318; *P*=0.0002; mean difference, 1.437; 95% CI, 0.426–2.448; *P*=0.0052). Subgroup analysis indicated differences in arterial stiffness between anthracycline‐based and non‐anthracycline‐based therapies (standard mean difference, 0.20; 95% CI, 0.001–0.41; *P*=0.048), but not follow‐up time.

**Conclusions:**

Significant arterial stiffening occurs following anticancer therapy. Our findings support the use of arterial stiffness as part of a targeted vascular imaging strategy for the identification of early cardiovascular injury during treatment and for the detection of long‐term cardiovascular injury into survivorship.

**Nonstandard Abbreviations and Acronyms**
Aod: aortic distensibility
CVD: cardiovascular disease
MD: mean difference
PWV: pulse wave velocity
SMD: standard mean difference

**Clinical Perspective**

**What Is New?**

- Exposure to anticancer therapy is associated with increased arterial stiffness in patients with cancer during treatment duration and continues years into survivorship.
- Patients with cancer with prior anticancer therapy exposure were found to have a 1.4 and 1.5 m/s increase in pulse wave velocity, a measure of arterial stiffness, on average when compared with healthy controls and pretreatment values, respectively.

**What Are the Clinical Implications?**

- Currently, there are no guidelines for monitoring vascular health in patients with cancer through the progression of treatment and into survivorship.
- Our findings support the use of noninvasive imaging strategies, such as pulse wave velocity, to monitor changes in vascular health and to assess the risk for development of cardiotoxicity with exposure to cancer treatments.
- Furthermore, these findings support the paradigm that there are global cardiovascular consequences that extend beyond the myocardium and the cardiovascular system should be considered as a whole when caring for a patient with cancer.

Anticancer treatments, including anthracyclines, alkylating agents, and vascular endothelial growth factor inhibitors, are associated with direct vascular damage^1^ and an increased risk of adverse vascular outcomes that can occur after the first treatment and persist into survival.^2^, ^3^, ^4^ As such, recent reports in vascular cardio‐oncology have highlighted the critical need to continuously monitor vascular health during treatment and into survivorship such that effective primary and secondary preventive strategies can be prescribed. However, although clinical monitoring for ventricular toxicities such as cardiomyopathy have been described (eg, echocardiography), there have been no systematic reports evaluating potential clinical strategies for monitoring vascular toxicity during and following anticancer treatment, reflecting a serious gap in our current knowledge and the need to identify potential imaging approaches.^5^, ^6^, ^7^

An increasingly recognized surrogate end point for cardiovascular disease (CVD) is local and regional measurements of arterial stiffness. In noncancer populations, arterial stiffness is independently predictive of all‐cause mortality and fatal/nonfatal cardiovascular outcomes and is used in CVD risk stratification.^8^, ^9^, ^10^, ^11^, ^12^, ^13^ We and others have demonstrated that patients with cancer and survivors exhibit increased arterial stiffness above the levels expected with aging alone.^2^, ^14^, ^15^ Therefore, because arterial stiffness is central to a comprehensive evaluation of vascular health and a surrogate end point for general CVD, the monitoring of arterial stiffness may serve as an important clinical approach in cancer populations receiving systemic anticancer therapies. However, the current evidence base is limited because of several factors, including small sample sizes, different measurement strategies, various study designs, and different durations of follow‐up. Therefore, to clarify these issues, we conducted the present systematic review and meta‐analysis with the primary aim to provide an overview of the current evidence for increases in arterial stiffness after anticancer therapy. A secondary aim was to examine whether changes in arterial stiffness differed as a function of follow‐up time and anticancer treatment type.

## METHODS

The authors declare that all supporting data are available within the article and its online supplementary files.

### Data Searches and Sources

Studies evaluating the relationship between arterial stiffness and anticancer therapy were retrieved from a systematic review of English literature in the Cochrane, PubMed, Google Scholar, and Web of Science databases until January 2019 by members of the research team (S.P., C.A.) with assistance from university research data informationists. The population search terms included “cancer,” “chemotherapy,” and “cardiotoxicity”; the descriptor search terms were “arterial stiffness,” “pulse wave velocity,” and “augmentation index.” Data sources were also identified through manual searches of references in the articles. All search results were downloaded to a research management system (Endnote, Clarivate Analytics) where data extraction began by removing duplicates, review articles, and letters to the editor. All remaining results underwent a full‐text review to determine eligibility in the analysis. The literature search and selection of studies was done by 2 independent reviewers (C.A., S.P.), and disagreements were resolved by consensus. This analysis was conducted in accordance with the Preferred Reporting Items for Systematic Reviews and Meta‐Analyses and registered at the International Prospective Register of Systematic Reviews (PROSPERO ID:150246, registration not published yet).

### Study Eligibility

Studies were considered eligible if they met the following criteria: (1) full‐length publication in a peer‐reviewed journal, (2) evaluated arterial stiffness via pulse wave velocity and or aortic/carotid stiffness, and (3) reported anticancer drugs used to treat cancer that have previously been associated with long‐term CVD risk.^16^ Because of the nature of our research question, the effect of anticancer therapy on arterial stiffness could be assessed via either longitudinal comparison of pretreatment baseline to posttreatment follow‐up or comparison of an anticancer treatment group to that of a healthy group matched in age, sex, and CVD risk factor. Exclusion criteria were studies lacking sufficient information on anticancer treatment or cancer type, longitudinal studies lacking sufficient information on baseline patient characteristics and stiffness data, and case‐control studies where the control group had a history of anticancer therapy. No restriction criteria were imposed regarding type of cancer, follow‐up time, or population age as a result of the limited number of studies.

### Modalities Included in the Study

**Figure 1:**
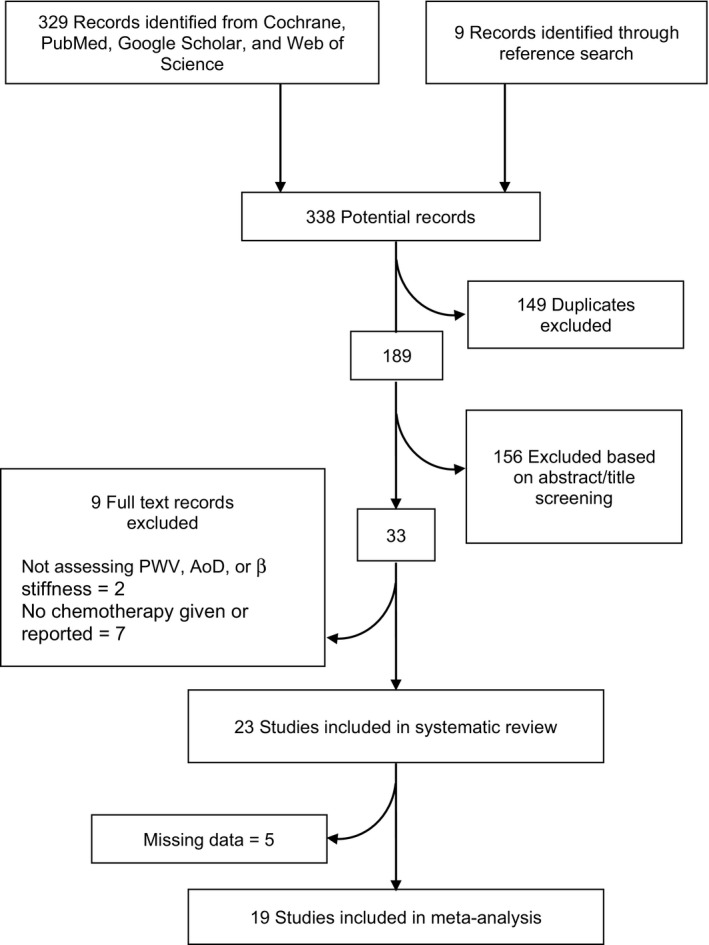
Flow chart of selection process of eligible studies. Flow through of the identification and selection of studies included in the systematic review and meta‐analysis. Aod indicates aortic distensibility; and PWV, pulse wave velocity.

We included 3 separate modalities in our analyses that evaluated arterial stiffness both as a local measurement of specific vessels via aortic distensibility (AoD) and β‐stiffness index (carotid artery and aorta) and regional assessments of pulse wave velocity (PWV) that gives an index of global arterial health (aortic, carotid–femoral and brachial–ankle). In our literature search, PWV, AoD, and β‐stiffness index were the most commonly reported and more important are validated measurements for assessing arterial stiffness.^17^, ^18^, ^19^ PWV is reported in meters per second and is calculated as the ratio of the distance between 2 points and the time taken to reach those 2 sites.^17^ AoD is calculated by the change in cross‐sectional area relative to the changes in arterial pressure, and the β‐stiffness index is calculated as the ratio of changes to relative changes in pressure and diameter.^19^ Figure 1 provides a visual representation of how arterial stiffness was measured in the studies included in our meta‐analysis.

### Extraction of Data

Data were extracted by 2 authors (C.A., S.P.) according to the Preferred Reporting Items for Systematic Review and Meta‐analyses statement^20^; discrepancies were resolved by consensus. For each study, we obtained population characteristics, measures of arterial stiffness, follow‐up duration, underlying malignancy, cancer treatment used, treatment duration, and reported measures of arterial stiffness via PWV, AoD, or β‐stiffness index. Some studies reported multiple measures of arterial stiffness; we agreed to extract PWV if reported because it is the gold standard for measuring arterial stiffness. The 2 authors agreed on consensus for extraction if both β stiffness and distensibility were reported. Upon review, 2 study design types were identified in the search results: longitudinal and cross‐sectional. Risk of bias was evaluated with the Newcastle–Ottawa Scale.^21^ Briefly, the quality of each study was determined using questions that assessed the categories of bias: selection, comparability, and exposure. No study was excluded on the basis of quality alone.

### Statistical Analysis

All statistical analyses were performed using the software R (version 3.5.1) with package meta. The effect sizes were calculated using the inverse variance method. Heterogeneity was evaluated using the Cochran Q test and Higgin I^2^ statistic. After examining the Cochran Q test and I^2^ statistic, significant heterogeneity was revealed, so we proceeded with a random effects model to minimize bias.^22^ We performed both a standard mean difference (SMD) analysis to account for different methods used to measure arterial stiffness and mean difference (MD) analysis to examine overall difference in PWV longitudinally in patients with cancer through treatment and in patients with cancer compared with cancer‐free controls. We felt it was appropriate to conduct both SMD and MD analyses to fully summarize the current literature and provide clinically relevant insights. Using the SMD method allowed us to include various methods used to evaluate arterial stiffness (PWV, AoD, β), and MD allowed us to determine the mean effect of changes in arterial stiffness measured by PWV, the current clinical gold standard. For the SMD analysis evaluating longitudinal changes in arterial stiffness, we had to correct differences in scaling (ie, increases in PWV indicate increases in stiffness, decreases in AoD indicate increases in stiffness). This was done by multiplying the mean values by −1 as directed by the Cochrane handbook for SMD meta‐analyses.^23^ The forest plots are provided with the SMD and MD with respective CIs for the comparison between previous research. The primary analysis evaluated the association between anticancer therapy and arterial stiffness, regardless of type of anticancer drug or follow‐up time. Subgroup analysis investigated whether differences in arterial stiffness differed as a function of follow‐up time (<6 months versus 6–12 months, 6–12 months versus >12 months, and <6 months versus >12 months following last anticancer treatment) and anticancer treatment type (anthracycline based versus non‐anthracycline‐based treatments). In addition, we performed a sensitivity analysis because of our high heterogeneity score with each analysis to determine if any one study was driving the results of the analyses.^22^ The sensitivity analysis was performed by calculating the pooled treatment effect of the studies that measured stiffness by PWV (ie, excluding studies that used AoD or carotid β stiffness) after excluding each study one at a time and calculating the SMD and MD. The treatment effect was considered significant if *P*<0.05.

## RESULTS

### Systematic Review

**Figure 2:**
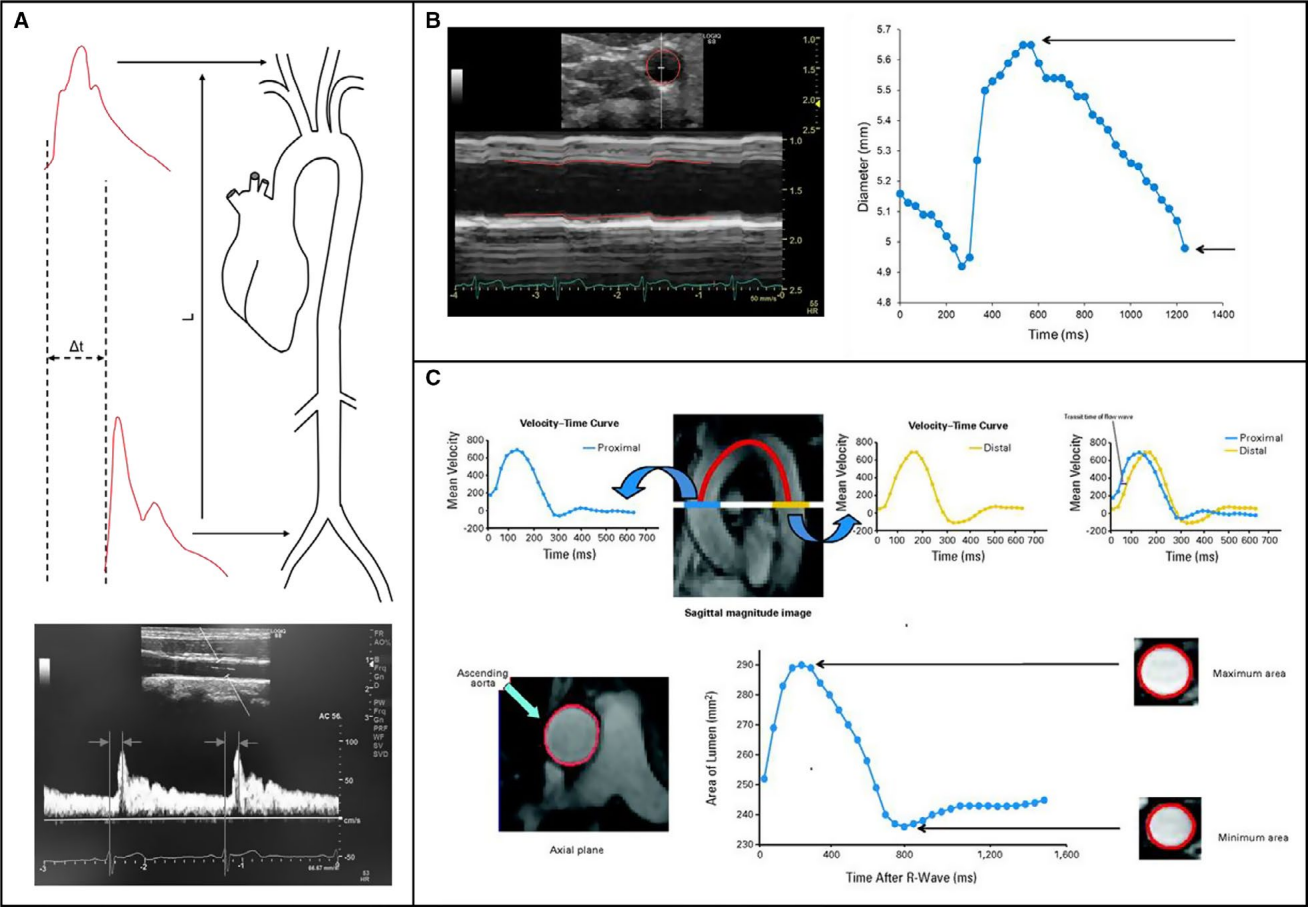
Determination of arterial stiffness. **A**, Pulse wave velocity can be calculated by dividing the distance (L) between 2 arterial sites by the difference in transit time (Δt) of pressure wave obtained via applanation tonometry or velocity wave obtained via Doppler ultrasonography (illustrated here) arrival between those sites. **B**, Carotid β stiffness can be calculated from B‐mode and M‐mode visualizations of the common carotid artery. From this image, maximal and minimal carotid diameters over the cardiac cycle can be determined by tracing the region of interest (red boundaries). **C/Upper**, Pulse wave velocity can be calculated from phase‐contrast cardiovascular magnetic resonance images of the aorta by dividing the distance between the ascending and descending thoracic aorta by the transit time of the flow wave computed on the basis time difference of the velocity–time curve at 2 different regions (blue line). **C/Lower**, Aortic distensibility can be calculated from phase contrast cardiovascular magnetic resonance imaging of the thoracic aorta. From these images, maximum and minimum aortic areas over the cardiac cycle can determined by tracing the region of interest (red boundaries). **C**, Reprinted from Chaosuwannakit et al^30^ with permission. Copyright ©2010, American Society of Clinical Oncology.

Our search identified 338 publications, which was narrowed by preliminary review to 189 after removing duplicates (Figure 2). Articles were excluded as a result of anticancer therapy not given or reported or using a method to measure stiffness that was not PWV, AoD, or β‐stiffness index. A total of 24 studies measuring stiffness were eligible; of those, 6 were missing data, and the authors were contacted via email and 1 responded. In the final analysis, 12 studies^1^, ^14^, ^15^, ^24^, ^25^, ^26^, ^27^, ^28^, ^29^, ^30^, ^31^, ^32^ were considered longitudinal or cohort studies. Ten studies^2^, ^30^, ^31^, ^32^, ^33^, ^34^, ^35^, ^36^, ^37^, ^38^ were case‐control studies with controls matched in age, sex, and cardiovascular risk factor. Three studies^30^, ^31^, ^32^ included a cross‐over case‐control design. In total, the included studies analyzed 2147 subjects (1043 patients, 1104 controls), and all studies were published from 2008 to present.

### Association of Increased Arterial Stiffness Based on Drug Class

#### Anthracycline Exposure and Arterial Stiffness

Of the included studies, 14 assessed arterial stiffness in patients who received primary anthracycline chemotherapy.^*^ Of those, 9 assessed acute (<1 year) arterial stiffness from baseline to completion of treatment or during treatment, with a follow‐up time that ranged 1 to 9 months. All but 2 of these studies reported a significant increase in arterial stiffness (range, ≈1%–95%). We recently reported a ≈20% increase in carotid artery stiffness in patients currently receiving anthracycline chemotherapy compared with matched noncancer controls,^2^ which is similar to several reports of patients in the months following treatment.^26^, ^31^, ^36^ In addition, some investigations have reported >50% increases in arterial stiffness within 4 to 6 months following treatment. Chaosuwannakit et al^30^ demonstrated a 2‐fold increase in carotid–femoral PWV in just 4 months from baseline measurement and a 3‐fold increase compared with the control group. Similarly, Drafts et al^15^ reported a rapid increase in arterial stiffness in the first month of the monitoring period with a 51% increase during the course of the full 6 months after correcting for baseline blood pressure. Importantly, these ranges of arterial stiffness increase are similar to that reported in aging populations and those with atherosclerosis,^39^, ^40^ highlighting the potential clinical implications. Contrary to a majority of the identified studies, Mizia‐Stec et al^1^ found no differences in carotid–femoral PWV from baseline to 6 months following the last anthracycline treatment. However, half of the participants in that study were on cardiovascular‐related medications (36% beta blockers, 19% angiotensin converting enzyme inhibitors, 10% calcium channel blockers) or supplemented with tamoxifen (58% of patients), all of which are known to have positive vascular effects that could have neutralized detrimental vascular effects from anthracycline administration.^41^, ^42^, ^43^, ^44^ It is worth noting that acute anthracycline cardiotoxicity in the cardiomyocytes is resolved shortly after discontinuation of the drug, and there is an asymptomatic period before latent overt cardiotoxicity.^45^, ^46^ It is reasonable to hypothesize this is occurring in the vasculature, but more data are needed to determine if there is a latency period before overt vascular toxicity.

It is important to note the high variability in magnitude of the acute increase in arterial stiffness between the identified studies. Similar ages of patients and treatment paradigms suggest that additional factors may contribute to the magnitude of stiffening that occurs with anthracycline chemotherapy. Although unknown that this time, these factors may include a patient's baseline cardiovascular health, use of combination therapy, simultaneous treatment with cardiovascular medications, and measurement modality. Additional future work is needed to elucidate what underlying factors increase the risk for large increases in arterial stiffness with anthracycline chemotherapy. Physiologically, these acute changes could be attributed to numerous factors including endothelial dysfunction, altered smooth muscle tone, and changes in the extracellular matrix regulated by factors such as catabolic matrix metalloproteases.^47^ Importantly, Bai et al^48^ demonstrated in a preclinical model that an increase in aortic matrix metalloproteases within days of doxorubicin administration, thus highlighting the potential for significant vascular remodeling during and in the months following treatment.^48^

In addition to acute changes in arterial stiffness, most long‐term effects of arterial health have been reported in childhood cancer survivors. Our search included 4 studies in adults, adolescents, and children who were treated with anthracycline chemotherapy as young children, and all 4 reported significant increases in arterial stiffness when compared with age‐matched controls with a follow‐up time ranging from 1 to 20 years. Herceg‐Cavrak et al^34^ reported a 13% increase in aortic PWV in children and adolescents (range, 6–20 years old) treated with anthracycline chemotherapy compared with healthy sex‐matched and age‐matched controls with an average follow‐up time of 2 years following chemotherapy administration. Conversely, Krystal et al^35^ reported no differences in carotid–femoral PWV between 51 age‐matched and sex‐matched controls and 68 adolescent childhood cancer survivors with an average follow‐up time of 7 years from end of treatment. However, in a subgroup analysis, patients >18 years old had a 10% increase in carotid–femoral PWV compared with >18‐year‐old controls, suggesting that older childhood cancer survivors develop chronic changes in arterial stiffness 5 to 10 years following treatment. Finally, and most notably, Jenei et al^33^ reported a 3‐fold increase in β stiffness with a 10‐year follow‐up period in adolescent childhood cancer survivors when compared with age‐matched and sex‐matched controls, further indicating that alterations in vascular integrity persist years to decades following anthracycline chemotherapy. Use of radiation therapy is commonly prescribed in the treatment of breast cancer and has been shown to increase arterial stiffness in cancer survivors.^49^ Although it is feasible that patients with prior histories of radiotherapy could have augmented the changes seen in our analysis, only 17% of patients receiving primary anthracycline chemotherapy had a history of radiotherapy.

#### Anti‐Angiogenic Tyrosine Kinase Inhibitors

One study included in our analysis assessed arterial stiffness in patients receiving vascular endothelial growth factor tyrosine kinase inhibitors.^14^ Alivon et al^14^ reported an acute increase in PWV of 11% in the first 7 to 10 days of therapy administration and statistically significant increases up through the second visit; however, further significance was not observed in either the third or fourth visit. The authors suggested the lack of continued significance may be attributed to the development of hypertension (systolic blood pressure >140 and/or diastolic blood pressure >90) in 49% of patients treated with anti‐angiogenic drugs; thus, 30% of patients were prescribed calcium channel blockers to control blood pressure. Regardless, other cardiovascular measures such as carotid stiffness assessed by ultrasound remained significant throughout the study with an overall increase of 13% from baseline to the fourth visit. Similarly, patients receiving anti‐angiogenic tyrosine kinase inhibitors had a 9% increase in PWV 6 weeks into treatment.^50^ These findings suggest anti‐angiogenic tyrosine kinase inhibitors cause acute changes in arterial integrity leading to greater arterial stiffness; however, our search did not provide any insight on the long‐term effects of anti‐angiogenic tyrosine kinase inhibitors and the association of arterial stiffness.

#### Alkylating Agents

Two studies examined the association of alkylating agents and arterial stiffness.^29^, ^32^ Willemse et al^29^ reported no change in aortic PWV with measurements at baseline, 3 months, and 9 months follow‐up that could be attributed to a small sample size (n=19). In contrast, Sekijima et al^32^ reported a 10% increase in brachial–ankle PWV from baseline to 12 months posttreatment as well as changes in other cardiovascular measures. This suggests that arterial stiffness persists chronically and is prevalent up to a year posttreatment with alkylating agents.

### Meta‐Analysis

Two separate meta‐analyses were conducted to determine if increases in arterial stiffness were associated with exposure to anticancer therapy. We separately examined the association over time in patients from pretreatment baseline to follow‐up after treatment and between controls and patient groups with anticancer therapy exposure.

**Figure 3:**
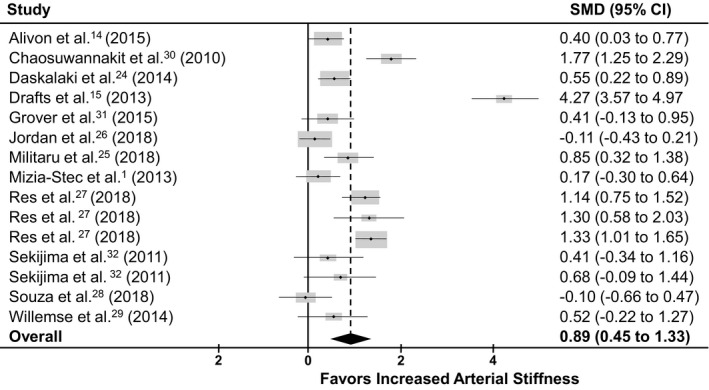
SMD results from longitudinal studies. Forest plot illustrating the effect size for each of the 12 longitudinal studies reporting arterial stiffness with anticancer chemotherapy. Overall effect favored greater arterial stiffness following anticancer treatment compared with pretreatment (SMD, 0.890; 95% CI, 0.447–1.332; *z*=3.95; *P*≤0.0001).^1^, ^14^, ^15^, ^24^, ^25^, ^26^, ^27^, ^28^, ^29^, ^30^, ^31^, ^32^ SMD indicates standard mean difference.

The first analysis included 12 longitudinal studies that examined patients with cancer receiving anticancer therapy from pretreatment baseline to follow‐up either during or after completion of treatment (Table 1).^1^, ^14^, ^15^, ^24^, ^25^, ^26^, ^27^, ^28^, ^29^, ^30^, ^31^, ^32^ The results revealed a statistically significant increase in arterial stiffness following anticancer therapy exposure in patients with cancer. Heterogeneity was confirmed with a statistically significant (*P*≤0.001) Q statistic (180.99, 305.25) and I² (92%, 96%) in both SMD and MD analyses, respectively. The random effects meta‐analysis revealed a significant increase in arterial stiffness after anticancer therapy in patients with cancer. This increase was seen in the months following chemotherapy compared with baseline values before the start of treatment (SMD, 0.890; 95% CI, 0.448–1.332; *z*=3.95; *P*≤0.0001; Figure 3)^1^, ^14^, ^15^, ^24^, ^25^, ^26^, ^27^, ^28^, ^29^, ^30^, ^31^, ^32^ (MD, 1.505; 95% CI, 0.789–2.221; *z*=4.12; *P*≤0.0001; Figure S1).^1^, ^14^, ^15^, ^25^, ^27^, ^28^, ^29^, ^30^, ^31^, ^32^

**Table 1:** Baseline to Follow‐Up

Study	Modality	Primary Chemotherapy	Cancer Type	Population (Sample Size, Age in y)	%Weight (SMD, MD)	Follow‐Up Duration (mo)	Results (Baseline vs Follow‐Up)	Risk of Bias Score (Max of 9)
Daskalaki et al (2014)^24^	AoD	Anthracycline	Lymphoma	N=70, 44±19	7.1%, N/A	>3 mo	2.48±0.2 vs 2.36±0.23^a^	9
Jordan et al (2018)^26^	AoD	Anthracycline	Breast, leukemia, lymphoma, sarcoma	N=76, 51±12	7.2%, N/A	6 mo	1.68±1.6 vs 1.86±1.6	8
Sekijima et al (2011)^32^	PWV PWV	Alkylating agent Alkylating agent	Ovarian Endometrial	N=14, 57±13 N=14, 57±8	6.1%, 4.8% 6.1%, 6.5%	12 mo 12 mo	14.67±2.88 vs 16.00±3.44^a^ 15.09±2.03 vs 16.67±2.45^b^	9
Willemse et al (2014)^29^	PWV	Alkylating Agent	Testicular	N=19, 20–54	6.1%, 9.5%	9 mo	4.6±0.7 vs 5.0±0.8	8
Chaosuwannakit et al (2010)^30^	PWV	Anthracycline	Breast, leukemia, Lymphoma	N=40, 52±11	6.7%, 6.6%	4 mo	6.9±2.3 vs 13.5±4.7^c^	8
Drafts et al (2013)^15^	PWV	Anthracycline	Breast, lymphoma, leukemia	N=53, 50±2	6.3%, 9.9%	6 mo	6.7±0.5 vs 10.1±1^d^	8
Grover et al (2015)^31^	PWV	Anthracycline	Breast	N=27, 54±11	6.7% 4.1%	4 mo	6.8±3.2 vs 8.9±6.4^b^	8
Militaru et al (2018)^25^	PWV	Anthracycline	Leukemia	N=30, 47±13	6.7%, 9.5%	6 mo	7.03±1.07 vs 7.97±1.12^d^	9
Mizia‐Stec et al (2013)^1^	PWV	Anthracycline	Breast	N=35, 50±9	6.9%, 1.8%	9–12 mo	16.7±11.8 vs 14.9±8.4	7
Souza et al (2018)^28^	PWV	Anthracycline	Breast	N=24, 52±9	6.6%, 9.2%	>3 mo	7.61±1.21 vs 7.49±49	9
Alivon et al (2015)^14^	PWV	Antiangiogenic tyrosine kinase inhibitor	Renal, liver, thyroid, melanoma, sarcoma	N=57, 59±15	7.1%, 8.4%	7–10 d	10±2.3 vs 11.1±3.1^a^	8
Res et al (2018)^27^	PWV	Antiangiogenic tyrosine kinase inhibitor	Kidney Gastrointestinal Bowel	N=60, 58±10 N=18, 67±7 N=93, 65±11	7.0%, 9.9% 6.2%, 9.8% 7.2%, 10.0%	N/A N/A N/A	7.3±0.7 vs 8.1±0.7^c^ 7.4±0.6 vs 8.2±0.6^c^ 7.6±0.6 vs 8.4±0.6^c^	8

AoD indicates aortic distensibility (mm Hg^−1^); Max, maximum; MD, mean difference; N/A, not available; PWV, pulse wave velocity (m/s); and SMD, standard mean difference.

a Indicates significant increases in arterial stiffness (*P*<0.01).

b Indicates significant increases in arterial stiffness (*P*<0.05).

c Indicates significant increases in arterial stiffness (*P*<0.0001).

d Indicates significant increases in arterial stiffness (*P*<0.001).

**Figure 4:**
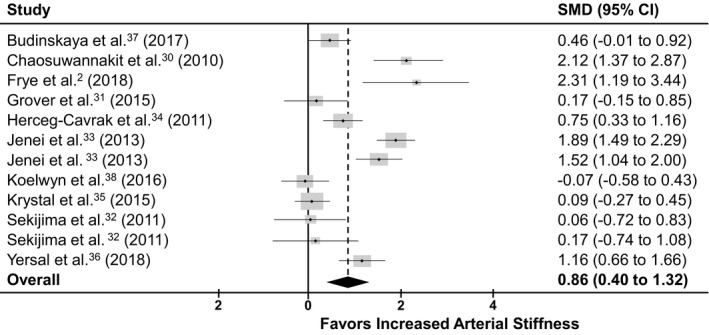
Standard mean difference results from cross sectional studies. Forest plot illustrating the effect size for each of the 10 cross‐sectional studies reporting arterial stiffness with anticancer chemotherapy. Overall effect favored greater arterial stiffness following anticancer treatment compared with matched healthy control participants (standardized mean difference, 0.860; 95% CI, 0.402–1.318; *z*=3.68; *P*=0.0002).^2^, ^30^, ^31^, ^32^, ^33^, ^34^, ^35^, ^36^, ^37^, ^38^

The second analysis included 10 cross‐sectional studies that examined the differences in arterial stiffness between patients with cancer with a history of anticancer therapy and control subjects matched in age, sex, and cardiovascular risk factor (Table 2).^2^, ^30^, ^31^, ^32^, ^33^, ^34^, ^35^, ^36^, ^37^, ^38^ The results revealed a statistically significant increase in arterial stiffness in the group of patients with cancer with prior anticancer therapy exposure. Heterogeneity of the analysis was confirmed with a statistically significant (*P*≤0.001) Q statistic (94.59, 132.74) and I² (88.4%, 94.0%) for the SMD and MD analyses, respectively. The random effects meta‐analysis revealed that arterial stiffness was significantly greater in cancer survivors treated with anticancer therapy than in healthy controls (SMD, 0.860; 95% CI, 0.402–1.318; *z*=3.68; *P*=0.0002; Figure 4)^2^, ^30^, ^31^, ^32^, ^33^, ^34^, ^35^, ^36^, ^37^, ^38^ (MD, 1.437; 95% CI, 0.426–2.448; *z*=2.79, *P*=0.0052; Figure S2).^30^, ^31^, ^32^, ^34^, ^35^, ^36^, ^37^, ^38^

**Table 2:** Patient Versus Control

Study	Modality	Primary Chemotherapy	Cancer Type	Patient Population (Sample Size, %W)	Patient Age (y)	Controls (Sample Size, %W)	Control Age (y)	Results (Patient vs Control)	Risk of Bias Score (Max of 9)
Frye et al (2018)^2^	ß stiffness index	Anthracycline	Breast, lymphoma, pancreatic, prostate	N=11, 6.2%	56±2	N=11, 6.2%	57±4	8±0.8 vs 6.3±0.6^a^	8
Jenei et al (2013)^33^	ß stiffness index ß stiffness index	Alkylating agent Anthracycline	Leukemia, lymphoma Leukemia, lymphoma	N=29, 8.9% N=67, 9.2%	14±5 15±4	N=72, 8.9% N=72, 9.2%	15±5 15±5	4.12±2.32 vs 2.08±0.6^b^ 6.45±3.25 vs 2.08±0.6^b^	8
Sekijima et al (2011)^32^	PWV PWV	Alkylating agent Alkylating agent	Ovarian Endometrial	N=14, 7.7% N=14, 7.1%	57±13 57±8	N=12, 7.7% N=7, 7.1%	55±11 57±5	16.0±3.44 vs 15.26±2.24^b^ 16.7±2.44 vs 16.18±3.56^a^	9
Budinskaya et al (2017)^37^	PWV	Anthracycline	Leukemia, lymphoma	N=21, 9.0%	19–24	N=122, 9.0%	19–24	7.4±1.08 vs 6.98±0.88^a^	4
Chaosuwannakit et al (2010)^30^	PWV	Anthracycline	Breast, lymphoma, leukemia	N=40, 7.8%	52±11	N=13, 7.8%	53±11	13.5±4.7 vs 4.6±0.9^c^	8
Grover et al (2015)^31^	PWV	Anthracycline	Breast	N=27, 8.1%	54±11	N=12, 8.1%	54±13	8.9±6.4 vs 7.9±4.0^a^	8
Herceg‐Cavrak et al (2011)^34^	PWV	Anthracycline	Lymphoma, sarcomas	N=53, 9.1%	14±4	N=45, 9.1%	12±3	6.24±1.34 vs 5.42±0.69^d^	5
Koelwyn et al (2016)^38^	PWV	Anthracycline	Breast	N=30, 8.8%	61±7	N=30, 8.8%	62±8	7.75±1.78 vs 7.78±1.47	7
Krystal et al (2015)^35^	PWV	Anthracycline	Lymphoma, leukemia, sarcomas	N=68, 9.3%	17±6	N=51, 9.3%	19±6	5.74±1.1 vs 5.65±1.88	7
Yersal et al (2018)^36^	PWV	Anthracycline	Breast	N=45, 8.8%	53±9	N=30, 8.8%	50±11	7.3±1.2 vs 5.8±1.4^d^	4

ß‐stiffness index (U). Max indicates maximum; and PWV, pulse wave velocity (m/s).

a Indicates significant increases in arterial stiffness (*P*<0.05).

b Indicates significant increases in arterial stiffness (*P*<0.01).

c Indicates significant increases in arterial stiffness (*P*<0.0001).

d Indicates significant increases in arterial stiffness (*P*<0.001).

Table 3 outlines the subgroup analyses of several different treatment effect modifiers within the studies. Treatment modifiers included type of chemotherapy consisting of anthracycline groups and nonanthracycline groups (tyrosine kinase inhibitors, alkylating agents) and time points of <6 months, 6 to 12 months, and >12 months of exposure to anticancer therapy. We sorted the groups of patients with cancer and control groups from all 19 studies into the appropriate treatment‐modifier groups and time‐modifier groups. A statistically significant difference in arterial stiffness was found between both chemotherapy‐treatment modifiers and each time point versus the corresponding control group (ie, anthracycline versus control, nonanthracycline versus control, <6 months versus control, 6–12 months versus control, >12 months versus control). There were no statistically significant differences in arterial stiffness found between the studies at different time points (<6 months versus 6–12 months, <6 months versus >12 months, 6–12 months versus >12 months). However, a significant difference in arterial stiffness was observed between the chemotherapy‐modifier groups of anthracycline versus nonanthracycline comparison groups (SMD, 0.20; 95% CI, 0.001–0.41; *P*=0.048). Forest plots for the time point and drug comparisons against control groups can be found in Figure S3.^1^, ^2^, ^14^, ^15^, ^24^, ^25^, ^26^, ^27^, ^28^, ^29^, ^30^, ^31^, ^32^, ^33^, ^34^, ^35^, ^36^, ^37^, ^38^

**Table 3:** Treatment Effect Modifiers

Treatment Modifier	No. of Studies	No. of Patients by Arm		SMD^a^			
				Between Patient and Control Arms		Between Modifier Subgroups^b^	
		Patient	Control	Mean (95% CI), Direction	*P* Value	Mean (95% CI), Direction	*P* Value
Time							
<6 mo	8	373	366	1.01 (0.28–1.73)	0.0064	−0.17 (−0.52 to 0.17), <6 mo vs 6–12 months	0.33
6–12 mo	4	87	90	0.69 (0.12–1.26)	0.0173	0.30 (−0.04 to 0.65), 6–12 mo vs >12 mo	0.09
>12 mo	6	313	422	0.83 (0.27–1.38)	0.0036	0.13 (−0.09 to 0.36), <6 mo vs >12 mo	0.25
Chemotherapy							
Anthracycline	15	635	701	0.94 (0.48–1.40)	<0.0001	0.20 (0.001 to 0.41), anthracycline vs nonanthracycline	0.048
Nonanthracycline	6	310	349	0.81 (0.43–1.19)	<0.0001		

SMD indicates standard mean difference.

a Represents the SMD between patient and modifier subgroup (eg, patient vs control <6 months into treatment, anthracycline group vs control).

b Represents the SMD between modifier subgroups (eg, anthracycline vs nonanthracycline groups, <6 months vs 6–12 months).

### Sensitivity Analysis

To ensure reliability of the present meta‐analyses with our high scores of heterogeneity, we performed a sensitivity analysis to evaluate the robustness of our SMD and MD. The one‐by‐one removal of studies revealed significance in a random effect model that was maintained through the entire analysis. Sensitivity analysis showed that the SMD and MD did not vary substantially with the exclusion of any one study.

## DISCUSSION

The present systematic review and meta‐analysis represent the most recent and updated work summarizing the evidence for increases in arterial stiffness in patients with cancer receiving anticancer therapy, which has previously been hypothesized as one of several major contributing factors for the increased risk of premature CVD in this population.^3^, ^49^ Overall, the meta‐analysis determined that patients with cancer have significantly increased arterial stiffness after anticancer therapy. In addition, subgroup analyses revealed that arterial stiffness is increased at all follow‐up time points and in response to both anthracycline and nonanthracycline treatment groups. This is the first systematic review and meta‐analysis to demonstrate this significant relationship between increased arterial stiffness and treatment with anticancer therapy. The clinical implications of these findings are several fold. First, these findings expand our understanding of the effects of anticancer therapy on the cardiovascular system beyond the heart by demonstrating that increases in arterial stiffness are detectable early after treatment and persists years into survivorship. This is significant given that a small increase in arterial stiffness in the general population increase the risk of CVD by >10%.^12^ Second, the results support the use of arterial stiffness as part of a targeted vascular imaging strategy that, based on its known association with CVD outcomes, can be used for the stratification of patient risk, identification of early cardiovascular injury during treatment, and detection of long‐term cardiovascular injury into survivorship.

The present study showed that anticancer therapy is associated with an increase in arterial stiffness, supporting the concept that anticancer therapy–induced cardiotoxicity extends beyond the left ventricle with direct vascular damage.^4^, ^51^ Several recent reviews have highlighted the importance of arterial stiffness in the evaluation of cardiovascular health in the general and non‐cancer‐patient populations, particularly for the prediction of all‐cause cardiovascular outcomes.^52^ Both local and regional assessments of arterial stiffness are significantly associated with an increased risk of developing various adverse cardiovascular outcomes.^9^, ^53^ Beyond its predictive capabilities, arterial stiffness has been shown to be directly associated with left ventricular dysfunction, left ventricular hypertrophy, and heart failure over time.^49^, ^54^ The stiffening of large arteries causes early return of peripheral reflection waves that augments late systolic pressure rather than early diastolic pressure; this limits coronary perfusion and increases myocardial oxygen demand.^55^ Thus, the overall importance of arterial stiffness as it directly relates to both overall cardiovascular health and changes in left ventricular mechanics has made it a parameter of interest that provides clinical insight beyond traditional risk factors such as aging, systematic coronary risk evaluation, and Framingham risk score^11^, ^17^, ^56^ and may provide a clinical tool for the monitoring of late‐developing cardiovascular outcomes.

In our literature search, we came across various methods of measuring arterial stiffness, including both local and regional measurements of arterial stiffness and measures of compliance, distensibility, and elasticity. In the present study, we included measures of regional stiffness (PWV) and local measurements (AoD, β), all of which have been shown to be associated with the manifestation of CVD.^57^ PWV is a direct measure of stiffness that records the speed of the pulse wave as it travels down the arterial tree, thus encompassing both large arteries and small muscular arteries^58^ and is considered the gold standard for measuring arterial stiffness.^17^, ^59^ The β‐stiffness index and AoD also provide a direct measure of arterial stiffness by measuring the changes in local pressure and arterial diameter in areas that are likely to develop atherosclerotic lesions.^54^ Although there is some potential for variability between local and regional measurements and within those that are pressure/volume related, 6 studies^1^, ^14^, ^27^, ^30^, ^31^, ^33^ included multiple measurements of arterial stiffness. Notably, Alivon et al^14^ reported significant increases in local measures of carotid β‐stiffness index, carotid distensibility, and a regional measurement with carotid–femoral PWV. These values remained significant after an adjustment for blood pressure and add to the rigor and reproducibility of these measurements with this specific population.

Exact pathophysiological mechanisms for increased arterial stiffness following anticancer chemotherapy are currently not known; however, we speculate that many of the same mechanisms contributing to arterial stiffness in response to aging and various types of CVD^60^, ^61^ are also occurring in patients with cancer receiving systemic anticancer therapy. Both normal aging and CVD progression are associated with vascular matrix remodeling and endothelial dysregulation of vascular smooth muscle tone as a result of increases in oxygen‐free radicals and the overexpression of inflammatory cytokines.^60^, ^62^, ^63^ Importantly, anthracyclines, tyrosine kinase inhibitors, and alkylating treatments have all been shown to directly or indirectly promote an intracellular oxidant to antioxidant imbalance, thereby eliciting oxidative stress.^64^, ^65^, ^66^ Within the vascular endothelium, nitric oxide control of vascular smooth muscle is decreased in response to elevations in oxidative stress.^67^, ^68^ In addition, oxidative stress causes intracellular damage to the endothelium and vascular smooth muscle layers through DNA damage, lipid peroxidation, and alteration of key cellular signaling pathways. These changes induce inflammation, necrosis, and apoptosis if damage is significant enough.^69^, ^70^ Together, oxidative injury coupled with increased inflammatory cytokines also leads to an abnormal production of collagen and depressed production of normal elastin. Such alterations in the balance of these vascular structural proteins causes a loss of elasticity and arterial stiffening.^47^ These mechanistic possibilities will require further investigation to determine whether they are relevant in the context of chemotherapy‐associated arterial stiffening.

It is also well established that arterial blood pressure can significantly impact measurements of arterial stiffness and must therefore be considered with interpretation of the changes in arterial stiffness reported in the present analysis.^71^ Importantly, Drafts et al^15^ demonstrated that patients with a higher systolic pressure at baseline had a faster increase in arterial stiffness, assessed via PWV, compared with those with lower pressures. This is a critical finding that highlights the integrative nature of arterial pressure and changes in stiffness and the importance of considering both physiological outcomes in the patient with cancer receiving anticancer therapy. However, in the present analysis, several studies corrected for blood pressure,^14^, ^24^, ^35^, ^37^ and all but 2 studies reported no change in arterial pressure with therapy.^36^, ^38^ In addition, the risk of treatment‐induced hypertension is primarily limited to drugs inhibiting the vascular endothelial growth factor signaling pathway,^16^, ^72^ which was used in only 2 of the studies included in the analysis.^14^, ^27^ Of those, Alivon et al^14^ adjusted for changes in pressure, and Res et al^27^ reported no changes in pressure. Although this does not exclude the possible confounding effects of small changes in pressure on the changes in stiffness observed, it does suggest that other factors may be at play. Future prospective investigations are needed to further evaluate the relationship between changes in arterial stiffness as it relates pressure in those treated for cancer and their impact on clinical outcomes.

### Clinical Perspective

Increased arterial stiffness is relevant for patient prognosis as greater arterial stiffness is associated with all‐cause mortality and fatal/nonfatal cardiovascular outcomes (eg, myocardial infarction, stroke, revascularization, hypertension, and heart failure) and is thus increasingly used in CVD risk stratification models.^8^, ^9^, ^10^, ^11^, ^12^ In our meta‐analysis, anticancer therapy was associated with greater arterial stiffness compared with both pretreatment baseline and untreated controls. Importantly, the findings from our MD analysis have extensive clinical impact. Our analysis revealed a 1.5 m/s increase in PWV across treatment in patients (Figure S1) and a 1.4 m/s increase in PWV in survivors of cancer with a history of anticancer therapy when compared with cancer‐free controls (Figure S2). This is clinically significant because every 1 m/s increase in PWV has been reported to equate to an age‐adjusted, sex‐adjusted, and risk factor–adjusted 14%, 15%, and 15% increased risk in cardiovascular events, cardiovascular mortality, and all‐cause mortality, respectively,^12^ which is consistent with the reported increased CVD risk in this population.^3^ These findings fill a serious gap in knowledge needed for the development of evidence‐based guidelines for the surveillance of vascular damage.^5^, ^6^, ^7^ Similar to how direct cardiomyocyte damage and decreased cardiac function led to the development of clinical guidelines to direct surveillance of cardiac damage via various imaging strategies,^5^ the present study, coupled with reports of direct vascular damage, support the need for specific vascular monitoring. An important outcome of this study is that arterial stiffness, which is a simple, noninvasive, cost‐efficient, and reproducible measurement, is an approach that should be considered as part of recommended care in those at‐risk patients receiving cardiotoxic anticancer therapies. Patients with cancer receiving cardiotoxic therapies are innately considered a high‐risk group as many patients diagnosed with cancer have subclinical or overt clinical CVD. Measuring arterial stiffness before the initiation of treatment can serve as a cumulative index of vascular health as well as an assessment of risk for the development of cardiotoxicity, both during and following treatment, that goes beyond those provided by measurements of left ventricular ejection fraction alone.

Previous work has investigated potential therapeutic interventions to restore arterial elasticity in ageing populations and decrease stiffness in patient populations. Increased carotid artery distensibility and decreased β stiffness has been reported in middle/older aged men and women following moderate‐intensity and high‐intensity aerobic exercise interventions^73^, ^74^, ^75^ and antioxidant supplementation of vitamins C and E and inorganic nitrates^76^, ^77^ have been shown to decrease PWV in hypertensive populations and older adults with increased CVD risk. Pharmacological agents such as angiotensin converting enzyme inhibitors, statins, and angiotensin receptor blockers have been demonstrated to decrease arterial stiffness in hypertension and end‐stage renal disease.^41^, ^42^, ^43^, ^68^ These decreases are hypothesized to be attributed to decreased levels of oxidative stress and inflammatory cytokine production, enhanced nitric oxide bioavailability, and decreased blood pressure. Further studies are needed to investigate the therapeutic effects of exercise, antioxidant, and cardiovascular medications to determine the effects on anticancer therapy associated increases in arterial stiffness in cancer survivors.

### Study Limitations

There were 4 main limitations of the present meta‐analysis and systematic review. First, as discussed previously, potential methodological limitations include dependence on blood pressure and age. Only 4 of the included studies^14^, ^24^, ^35^, ^37^ were adjusted for systolic blood pressure, sex, and body mass index; however, most of the studies included controls or patients who were age, cardiovascular risk factor, and sex matched and who served as their own controls from the start of treatment to follow‐up. There were only 2 studies that reported a significant difference in blood pressure between controls and patient groups.^36^, ^38^ In the other 17 studies, there were no significant differences in blood pressure between patients and controls and longitudinally between baseline and follow‐up periods in patients with cancer as they received treatment. Therefore, blood pressure, although a critical confounding factor in determining arterial stiffness, appeared to have a minor role in the reported increases in arterial stiffness of the present analysis. Second, we could not control for variable drug combination and dosage between and within studies. Third, there is the potential that factors such as obesity, hypertension, and use of medications could have influenced arterial stiffness outside of anticancer therapy, although most studies measured changes over time, which would eliminate this potential limitation. Regardless, our analysis showed significant increases in arterial stiffness in various methods of measuring arterial stiffness and in patients on different combinations of anticancer drugs translating the potential use of arterial stiffness in clinical practice for a wider population than only those receiving known cardiotoxic chemotherapies. Lastly, we recognize some statistical limitations exist with using SMD in weighting studies, limitations in standardizing different modalities that measure arterial stiffness, the possibility of upweighting some individual studies, and high levels of heterogeneity. However, our sensitivity analyses maintained significance even with removing higher weighted studies. We also acknowledge the multitesting burden presented with our analyses because 3 studies contained both longitudinal and cross‐sectional data.

## CONCLUSIONS

The results of the present meta‐analysis show an associated increase in arterial stiffness in patients receiving anticancer therapy when compared with healthy age‐matched and sex‐matched controls and from baseline before treatment when compared over time during treatment or after completion of treatment. Local and regional arterial stiffness measurements have independent predictive ability in all‐cause mortality and cardiovascular events in various patient populations who share similar cardiovascular risk factors as patients with cancer receiving cardiotoxic anticancer therapy. These findings support the need to measure vascular health outside of monitoring changes in left ventricular function in this population through the course of treatment to monitor/prevent the onset of overt CVD.

## Sources of Funding

None.

## Disclosures

None.

## Supporting information


**Figures S1–S3 References 1, 8, 10, 13, 16, 19, 20, 22, 26–29, 39, 41, 53, 55, 60, 72, and 76**

## Note

1 References 1, 2, 15, 24, 25, 26, 28, 30, 33, 34, 35, 36, 37, 38.
