# Supplementary material for: Anticancer Therapy–Related Increases in Arterial Stiffness: A Systematic Review and Meta‐Analysis
Source: J Am Heart Assoc. 2020 Jul 10;9(14):e015598. doi: 10.1161/JAHA.119.015598 (PMC7660726; doi:10.1161/JAHA.119.015598)
Supplement: Supplementary file 1 — Figures S1–S3 References 1, 8, 10, 13, 16, 19, 20, 22, 26–29, 39, 41, 53, 55, 60, 72, and 76 [file JAH3-9-e015598-s001.pdf]

SUPPLEMENTAL MATERIAL

Figure S1. Mean Difference Results from Longitudinal Studies.

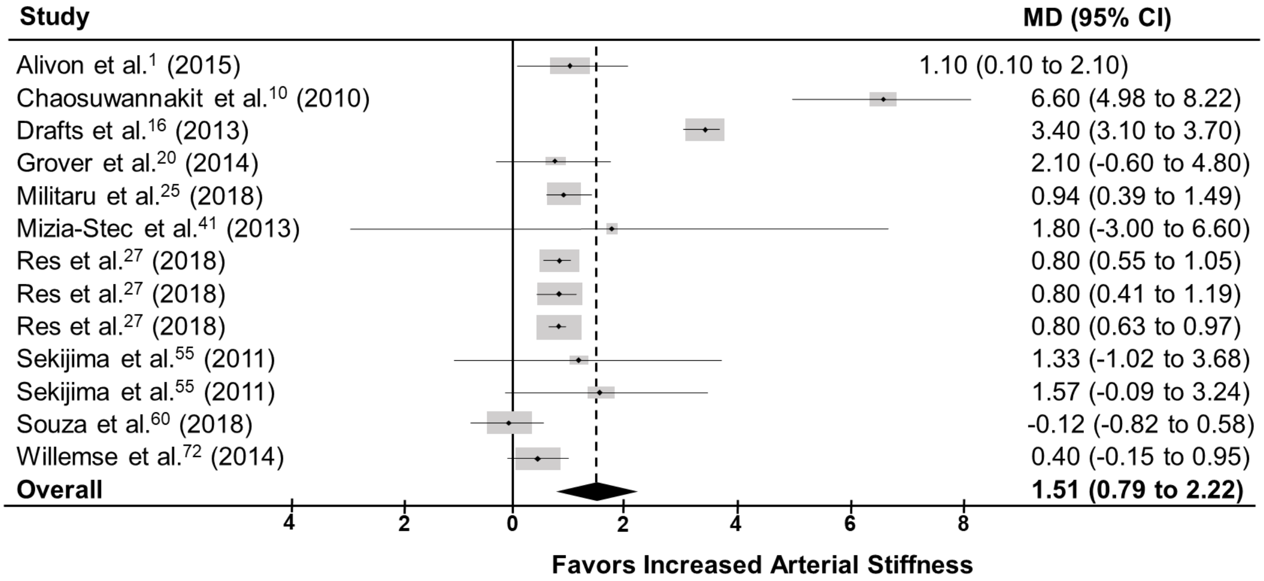

Forest plot illustrating the effect size for each of the 10 longitudinal studies reporting arterial stiffness with anticancer chemotherapy. Overall effect favored greater arterial stiffness following anticancer treatment compared to pre-treatment. (mean difference [MD]=1.505, 95% CI = 0.789-2.221, z= 4.12, $p \leq 0.0001$) [1, 10, 16, 20, 39, 41, 53, 55, 60, 72]

Figure S2. Mean Difference Results from Cross Sectional Studies.

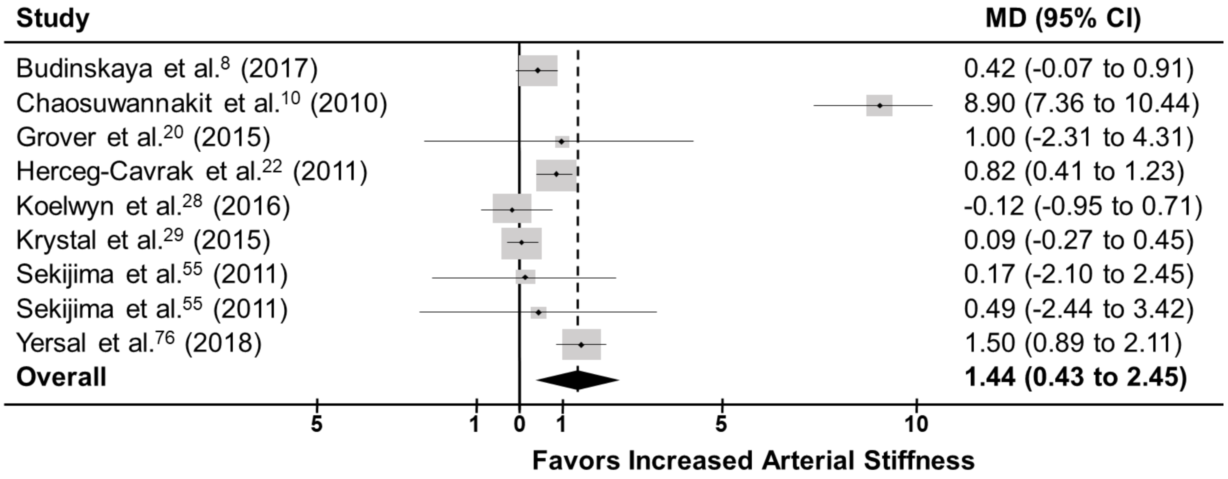

Forest plot illustrating the effect size for each of the 8 cross-sectional studies reporting arterial stiffness with anticancer chemotherapy. Overall effect favored greater arterial stiffness following anticancer treatment compared to matched healthy control participants. (mean difference [MD] = 1.437, 95% [CI] = 0.426-2.448, z= 2.79, p= 0.0052) [8, 10, 20, 22, 28, 29, 55, 76]

Figure S3. Standard Mean Difference Results from Subgroup Analysis.

Study or Subgroup	Standard Mean Difference	SD	Weight (%)	Standard Mean Difference	Random Effect, 95% CI
**Anthracycline Subgroup**					
Budinskaya et al.[8] (2017)	0.4486	0.2378	6.8%	0.46 (-0.01 to 0.92)	
Chaosuwannakit et al.[10] (2010)	1.7666	0.2655	6.7%	1.77 (1.25 to 2.29)	
Daskalaski et al.[13] (2013)	0.5538	0.1722	7.0%	0.55 (0.22 to 0.89)	
Drafts et al.[16] (2013)	4.2696	0.3564	6.3%	4.27 (3.57 to 4.96)	
Frye et al.[19] (2018)	2.3229	0.5743	5.1%	2.31 (1.19 to 3.44)	
Grover et al.[20] (2015)	0.4090	0.2752	6.7%	0.41 (-0.13 to 0.95)	
Herceg-Cavrak et al.[22] (2011)	0.7455	0.2098	6.9%	0.75 (0.33 to 1.16)	
Jenei et al.[26] (2012)	1.8923	0.2051	6.9%	1.89 (1.49 to 2.29)	
Jordan et al.[27] (2018)	0.1964	0.1815	7.0%	0.19 (-0.16 to 0.55)	
Koelwyn et al.[28] (2016)	-0.0726	0.2583	6.7%	-0.07 (-0.58 to 0.43)	
Krystal et al.[29] (2015)	0.0884	0.1853	7.0%	0.09 (-0.27 to 0.45)	
Militaru et al.[25] (2018)	0.8471	0.2703	6.7%	0.85 (0.32 to 1.37)	
Mizia-Stec et al.[41] (2013)	0.1738	0.2395	6.8%	0.17 (-0.29 to 0.64)	
Souza et al.[60] (2018)	-0.0951	0.2888	6.6%	-0.09 (-0.66 to 0.47)	
Yersal et al.[76] (2018)	1.1569	0.2548	6.7%	1.16 (0.66 to 1.66)	
Subtotal (95% CI)				0.94 (0.48 to 1.40)	
Heterogeneity Q=204.08, d.f.= 14 (p<0.0001); I^2 = 93.3%					
Test for overall effect: Z= 3.97 (p<0.0001)					
**Non-Anthracycline Subgroup**					
Alivon et al.[1] (2015)	0.3958	0.2035	13.0%	0.39 (-0.01 to 0.79)	
Jenei et al.[26] (2012)	1.5172	0.2454	12.2%	1.52 (1.04 to 1.99)	
Jordan et al.[27] (2018)	-0.3412	0.3564	10.0%	-0.34 (-1.04 to 0.36)	
Res et al.[53] (2018)	1.1356	0.1971	13.1%	1.14 (0.75 to 1.52)	
Res et al.[53] (2018)	1.3037	0.3709	9.7%	1.30 (0.58 to 2.03)	
Res et al.[53] (2018)	1.3279	0.1622	13.7%	1.33 (1.01 to 1.65)	
Sekijima et al.[55](2011)	0.4065	0.3824	9.5%	0.41 (-0.34 to 1.16)	
Sekijima et al.[55](2011)	0.6792	0.3904	9.4%	0.68 (-0.09 to 1.44)	
Willemse et al.[72] (2014)	0.5211	0.3805	9.5%	0.52 (-0.22 to 1.27)	
Subtotal (95% CI)				0.81 (0.43 to 1.19)	
Heterogeneity Q=36.85, d.f.= 8 (p<0.0001); I^2 = 78.3%					
Test for overall effect: Z= 4.19 (p<0.0001)					
**<6 Months Subgroup**					
Alivon et al.[1] (2015)	0.3958	0.2035	12.8%	0.40 (-0.01 to 0.79)	
Chaosuwannakit et al.[10] (2010)	1.7666	0.2655	12.4%	1.77 (1.25 to 2.29)	
Daskalaski et al.[13] (2013)	0.5538	0.1723	12.9%	0.55 (0.22 to 0.89)	
Drafts et al.[16] (2013)	4.2696	0.3564	11.8%	4.27 (3.57 to 4.97)	
Grover et al.[20] (2015)	0.4090	0.2753	12.4%	0.41 (-0.13 to 0.95)	
Jordan et al.[27] (2018)	0.1964	0.1623	13.0%	0.19 (-0.21 to 0.43)	
Militaru et al.[25] (2018)	0.8471	0.2703	12.4%	0.85 (0.32 to 1.38)	
Souza et al.[60] (2018)	-0.0951	0.2889	12.3%	-0.09 (-0.66 to 0.47)	
Subtotal (95% CI)				1.01 (0.28 to 1.73)	
Heterogeneity Q=141.45, d.f.= 7 (p<0.0001); I^2 = 95.1%					
Test for overall effect: Z= 2.73 (p=0.0064)					
**6-12 Months Subgroup**					
Frye et al.[19] (2018)	2.3129	0.5743	14.0%	2.31 (1.18 to 3.44)	
Mizia-Stec et al.[41] (2013)	0.1738	0.2395	25.6%	0.17 (-0.29 to 0.64)	
Sekijima et al.[55](2011)	0.4065	0.3824	20.2%	0.41 (-0.34 to 1.16)	
Sekijima et al.[55](2011)	0.6792	0.3904	19.9%	0.68 (-0.09 to 1.44)	
Willemse et al.[72] (2014)	0.5211	0.3806	20.3%	0.52 (-0.22 to 1.27)	
Subtotal (95% CI)				0.69 (0.12 to 1.26)	
Heterogeneity Q=12.08, d.f.= 4 (p=0.0167); I^2 = 66.9%					
Test for overall effect: Z= 2.38 (p=0.0173)					
**>12 Months Subgroup**					
Budinskaya et al.[8] (2017)	0.4586	0.2378	14.2%	0.46 (-0.01 to 0.92	
Herceg-Cavrak et al.[22] (2011)	0.7455	0.2099	14.5%	0.75 (0.33 to 1.16)	
Jenei et al.[26] (2012)	1.8923	0.2051	14.6%	1.89 (1.49 to 2.29)	
Jenei et al.[26] (2012)	1.5172	0.2454	14.1%	1.52 (1.03 to 1.99)	
Koelwyn et al.[28] (2016)	-0.0726	0.2583	13.9%	-0.07 (-0.58 to 0.43)	
Krystal et al.[29] (2015)	0.0884	0.1853	14.8%	0.09 (-0.27 to 0.45)	
Yersal et al.[76] (2018)	1.1569	0.2549	14.0%	1.16 (0.66 to 1.66)	
Subtotal (95% CI)				0.83 (0.27 to 1.38)	
Heterogeneity Q=67.11, d.f.= 6 (p<0.0001); I^2 = 91.1%					
Test for overall effect: Z= 2.91 (p=0.0036)					

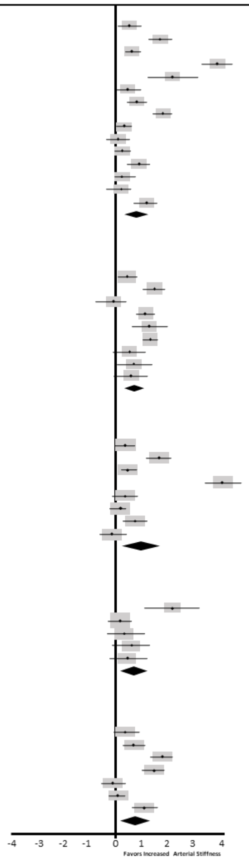

Forest plots illustrating the effect size for each subgroup analysis separated by time point and drug class. Overall effect for each analysis favored greater arterial stiffness with each drug class and all time points after treatment when compared to healthy control participants (Anthracycline subgroup vs. control, standard mean difference [SMD] = 0.94, 95% [CI] = 0.48-1.40, z = 3.97, p<0.0001; Non-anthracycline subgroup vs. control, [SMD] = 0.81, 95% [CI] = 0.43-1.19, z = 4.19, p<0.0001; <6months of treatment vs. control, [SMD] = 1.01, 95% [CI] = 0.28-1.73, z = 2.73, p=0.00064; 6-12 months treatment vs. control, [SMD] = 0.69, 95% [CI] = 0.12-1.26, z = 2.38, p=0.0173; >12 months treatment vs. control, [SMD] = 0.83, 95% [CI] = 0.27-1.38, z = 2.91, p=0.0036) [1, 8, 10, 13, 16, 19, 20, 22, 26-29, 39, 41, 53, 55, 60, 72, 76]
